# Acute and prolonged effects of anti-malarial drugs on mitochondrial respiration in atrial cardiomyocytes for cardiac safety evaluation

**DOI:** 10.1371/journal.pone.0351511

**Published:** 2026-09-11

**Authors:** Rahme Nese Safakli, Bonn Lee, Karan R. Chadda, Savithri Sathivelu, Ioannis Smyrnias, Christopher L.H. Huang, Joel Tarning, Cesare Terracciano, Gary Tse, Charlotte E. Edling, Kamalan Jeevaratnam

**Affiliations:** 1 Faculty of Health and Medical Science, School of Veterinary Medicine, University of Surrey, Guildford, United Kingdom; 2 Institute of Inflammation and Ageing, University of Birmingham, Birmingham, United Kingdom; 3 Homerton College, University of Cambridge, Cambridge, United Kingdom; 4 Royal Free London NHS Foundation Trust, London, United Kingdom; 5 Department of Physiology, Development and Neuroscience, Downing Site, University of Cambridge, Cambridge, United Kingdom; 6 Nuffield Department of Medicine, University of Oxford, Oxford, United Kingdom; 7 National Heart & Lung Institute, Imperial College London, London, United Kingdom; 8 School of Nursing and Health Sciences, Hong Kong Metropolitan University, Hong KongChina; Para Federal University, BRAZIL

## Abstract

**Objectives:**

Malaria is a global public health problem, causing significant morbidity and mortality, particularly in low and middle-income countries (LMICs). While mass drug administration (MDA) programs are central to elimination efforts, their effectiveness is limited by concerns over cardiotoxicity, resistance, and potential impacts on mitochondrial function. This study investigates the acute and prolonged effects of single and combination anti-malarial drugs on mitochondrial respiration in atrial cardiomyocytes.

**Methods:**

Human iPSC-derived atrial cardiomyocytes were treated with either single or combination anti-malarial drugs. Mitochondrial respiration was assessed using an extracellular flux analyser (Agilent Seahorse).

**Results:**

Treatment with mefloquine (MFQ) and its combinations reduced mitochondrial respiration, indicating impaired energy generation in cardiomyocytes. Prolonged exposure to halofantrine (HFN) significantly reduced maximal respiration and spare respiratory capacity, demonstrating compromised mitochondrial function. In contrast, acute and prolonged exposure to amodiaquine (AMD), artemether (ART), chloroquine (CQ), and piperaquine (PPQ) maintained mitochondrial function. While acute exposure to Ivermectin (IVM), an anti-parasitic drug used in malaria treatment programmes, did not affect mitochondrial health, prolonged exposure resulted in reduced coupling efficiency.

**Conclusions:**

These findings provide insights into the effects of anti-malarial drugs on mitochondrial function in cardiomyocytes. Further studies should aim to elucidate the molecular mechanisms underlying these drug-induced mitochondrial effects.

## Introduction

Malaria is a mosquito-borne parasitic disease which causes approximately 247 million clinical infections and results in an estimated 619,000 deaths annually, with the majority of cases occurring in low and middle-income countries (LMICs) [1]. Despite extensive global efforts, over 3.3 billion individuals remain at risk, and current prevention strategies are hindered by the evolutionary adaptation of both the parasite and its mosquito vector [1,2]. In addition to the significant public health burden, the cardiac safety of anti-malarial drugs is a major concern, particularly regarding cardiotoxicity and arrhythmic risk [3,4]. Anti-malarial drugs may disrupt mitochondrial function, affect cellular energy production and contribute to arrhythmias [4–6]. These drugs may lead to altered action potential, mitochondrial respiration, increased production of reactive oxygen species (ROS), and compromised calcium handling, all of which can contribute to impaired cardiac function and increase the risk of arrhythmias [3]. However, significant gaps remain in understanding their potential pro-arrhythmic effects and the underlying mechanisms, which need to be addressed before the widespread introduction of mass drug administration.

Cardiac complications in malaria cases may arise either from the pathophysiological effects of the infection itself or as adverse effects of anti-malarial treatments [3]. While reports indicate that cardiovascular complications are relatively rare, it is important to consider that approximately 90% of malaria-related deaths occur in resource-limited African regions, which may result in under-diagnosis and under-reporting [7]. Due to the development of resistance to single drug therapies, alternative treatments such as combination therapies have emerged as a strategy to improve efficacy and reduce the potential for resistance [8]. Combination therapies are designed to combine two or more bioactive agents. The World Health Organization (WHO) has approved the use of combination therapies, which are classified into two categories: artemisinin-based combination therapies (ACTs) and non-artemisinin-based therapies [9]. ACTs have been pivotal to the success of global malarial control efforts. However, the use of combination therapies raises concerns regarding potential drug-drug interactions, resistance, and compounded risks, including cardiac safety [3,9]. Thus, a major concern with the widespread use of both single anti-malarial drugs and the combination treatments is the potential cardiac complications. Several anti-malarial drugs, particularly quinoline derivatives and structurally similar compounds, have been linked to cardiac issues such as bradycardia (a resting heart rate of below 60 beats per minute (BPM)), hypotension, heart block, and various ECG abnormalities [4]. Moreover, anti-malarial drug chloroquine reported to cause significant disruption in the structure and function of mitochondria, resulting in the increased intracellular calcium (Ca^2+^), depolarisation of the mitochondrial membrane potential, and reactive oxygen species (ROS) production, both of which leads to proarrhythmic electrophysiological changes in cardiomyocytes [3,10,11]. Mitochondria generate most of the cellular energy required for contractile function through the process of oxidative phosphorylation. In addition, mitochondria are responsible for calcium homeostasis, ROS signalling, and the metabolism of nucleotides, amino acids, and lipids, as well as regulating cardiomyocyte survival [12–14]. Given the high energy demands of cardiomyocytes, mitochondrial dysfunction has profound effects on cellular electrophysiology. Mitochondria play a critical role in myocardial contraction by regulating intracellular Na^+^, K^+^, and Ca^2+^ levels [15,16]. Reduced ATP production impairs the activity of ion pumps such as the Na ⁺ /K ⁺ -ATPase and Ca² ⁺ -ATPase, leading to ionic imbalance, while increased ROS levels can modify ion channel function. Furthermore, disrupted mitochondrial calcium handling promotes intracellular Ca² ⁺ overload, which can trigger delayed afterdepolarisations [16,17]. Together, these alterations contribute to electrophysiological instability, thereby increasing the risk of cardiac arrhythmias and arrhythmia-related sudden cardiac death. Given the broad range of adverse effects linked to anti-malarial drugs, understanding how these anti-malarial drugs affect mitochondrial function is essential for evaluating their potential to disrupt cardiac cellular functions and ensuring their safety in widespread malaria prevention efforts.

**Fig 1:**
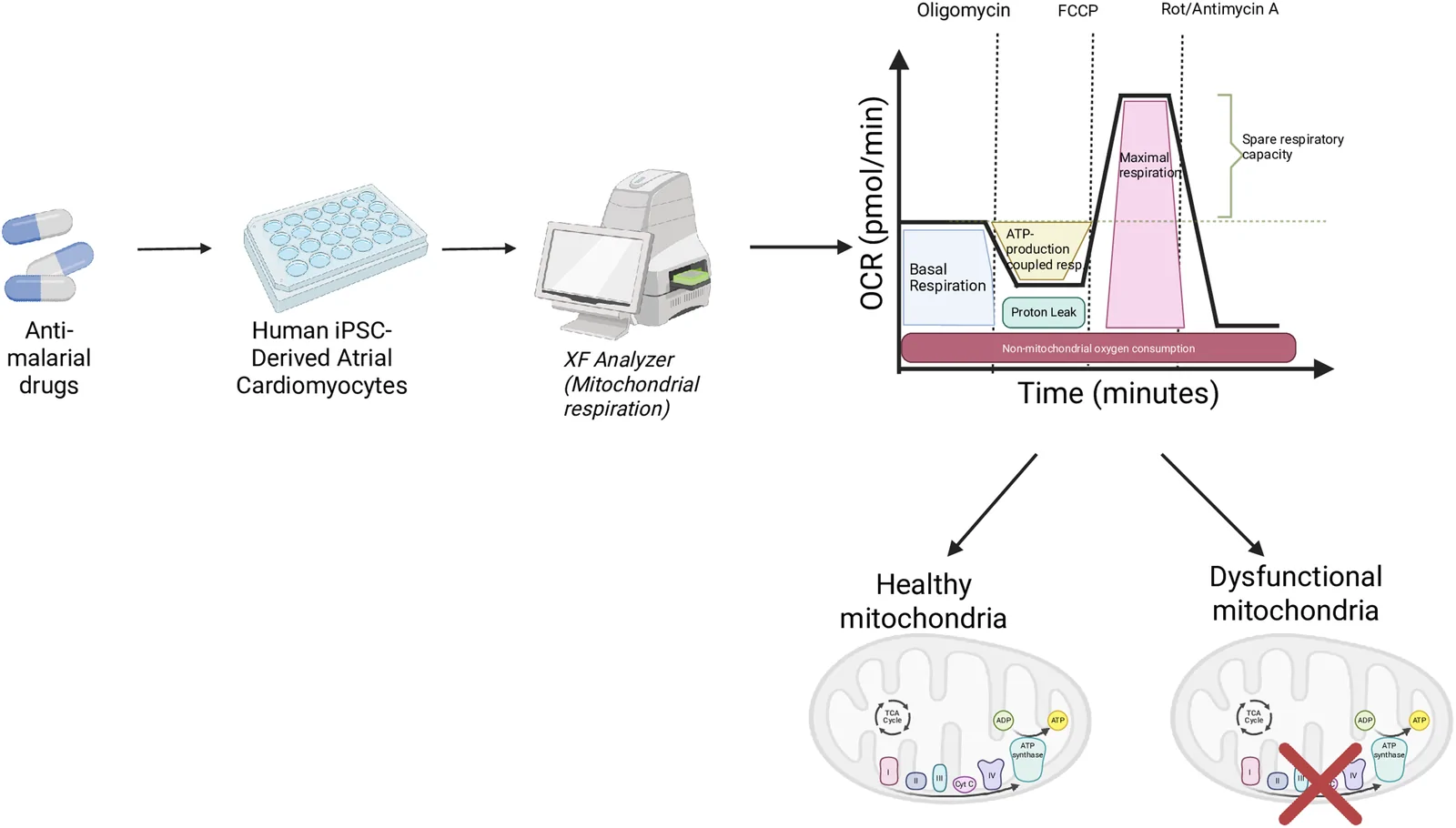
Graphical Abstract. Malaria remains a major health burden in low and middle-income countries (LMICs). Despite large-scale elimination efforts, challenges persist due to cardiotoxicity and drug resistance. This study investigates how widely used anti-malarial drugs, both as single agents and in combination therapies, affect mitochondrial bioenergetics in atrial cardiomyocytes, providing new insights into potential mechanisms of drug-induced cardiotoxicity and informing safer strategies for malaria treatment and prevention.

Therefore, in this study, we investigated the acute and prolonged effects of single and combination doses of anti-malarial drugs on mitochondrial respiration in atrial cardiomyocytes using an extracellular flux analyser (Fig 1).

## Materials and methods

### Human iPSC culture

Human induced pluripotent stem cells (iPSC)-derived atrial cardiomyocytes (axoCells, AXOL, UK) were cultured according to the manufacturer’s instructions. iPSCs (30,000 cells/well) were seeded on Seahorse XFe24 culture plates coated with 1X human fibronectin (AXOL, UK).

### Drug treatments

Following culturing, iPSCs were allowed to settle and beat for at least 5 days before any experimental manipulations were initiated. The in vitro drug concentrations used in this study were determined based on the therapeutic concentrations employed clinically, with reference to peak plasma (Cmax) data, and were applied according to total plasma concentrations. For combination therapies, the experiments were conducted using concentrations corresponding to those used in the fixed-dose formulations, and the experimental concentrations for the single agents were equal to those used in the combination therapies. The pharmacological data for each individual drug were referred to the following sources: dihydroartemisinin (DHA, 1.75 μM, [18], Cayman Chemical, USA) and piperaquine (PPQ, 0.5 μM, [19], Cayman Chemical, USA), both dissolved in DMSO. Chloroquine phosphate (CQ, 3 μM, [20], Sigma Aldrich, UK) was dissolved in ddH_2_O, while the following drugs were dissolved in methanol: artemether (ART, 0.7 μM, [21], Sigma Aldrich, UK), amodiaquine hydrochloride (AMD, 0.03 μM, [22], Sigma Aldrich, UK), halofantrine hydrochloride (HFN, 2 μM, [23], Sigma Aldrich, UK), mefloquine hydrochloride (MFQ, 4 μM, [24], Sigma Aldrich, UK), and ivermectin (IVM, 0.1 μM, [25], Sigma Aldrich, UK). Vehicle solutions were matched to the diluents and concentrations used for the drug treatments. No differences were observed between vehicle controls. iPSCs were exposed to the drugs for acute (90 minutes) and prolonged (24 hours) periods, after which mitochondrial respiration was assessed. The acute exposure was designed based on the minimum exposure time recommended by the manufacturer, while the prolonged exposure condition was set as 24 hours, referring to a previous study showing that mitochondrial respiration parameters such as proton leak and coupling efficiency undergo significant remodelling following 24 hours cellular stress exposure in cardiomyoblasts [26]. Moreover, 24 hours exposure duration is commonly used to evaluate rapid parasite killing kinetics and stage-specific antimalarial activity in Plasmodium falciparum in vitro assays [27].

### Mitochondrial respiration

The oxygen consumption rate (OCR) of iPSCs was measured using an XFe24 extracellular flux analyzer (Seahorse; Agilent, UK), following the manufacturer’s protocol for mitochondrial stress tests (Agilent, UK). Following treatments, the cell medium was replaced with Seahorse XF assay media supplemented with 1mM pyruvate, 2mM glutamine and 10mM glucose, and incubated for 30 minutes prior to running the assay. Basal oxygen consumption rate, Adenosine triphosphate (ATP) production-coupled oxygen consumption, and maximal oxygen consumption rate were determined using a mitochondrial stress test protocol involving sequential administration of oligomycin (1 µM), FCCP (2 µM), and rotenone/antimycin A (1 µM each).

Following the assay, iPSCs were lysed in RIPA extraction buffer (25mM Tris-HCl pH 7.4, 150mM NaCl, 5mM EDTA, pH set to 7.2, then 0.5–1% NP-40 or Triton X-100 were added) containing 1% protease inhibitor (Sigma, UK) and Phosphatase Inhibitor Cocktails 2 (Sigma, UK). The lysates were incubated on ice for 15 minutes and then centrifuged at 13,000 x g for 10 minutes at 4 °C. Following centrifugation, protein concentrations of the supernatants were measured using a Pierce BCA Protein Assay Kit, according to the manufacturer’s protocol (ThermoFisher, UK). Data were normalised to the protein concentration in each well.

### Statistical analysis

Data were analysed using GraphPad Prism software and are presented as mean ± SEM, unless otherwise specified. Statistical comparisons between relevant groups were conducted using Welch’s t-test or one-way ANOVA, as appropriate. All experiments included three biological replicates from three vials individually purchased from the manufacturer.

Post hoc ANOVA-based power analyses were performed on representative Seahorse parameters, including basal respiration and coupling efficiency, across experimental conditions to confirm adequate statistical sensitivity for detecting treatment-associated effects. Statistical significance was defined as p < 0.05.

## Results

### Acute exposure to the anti-malarial drug Mefloquine reduces mitochondrial respiration in atrial cardiomyocytes

**Fig 2:**
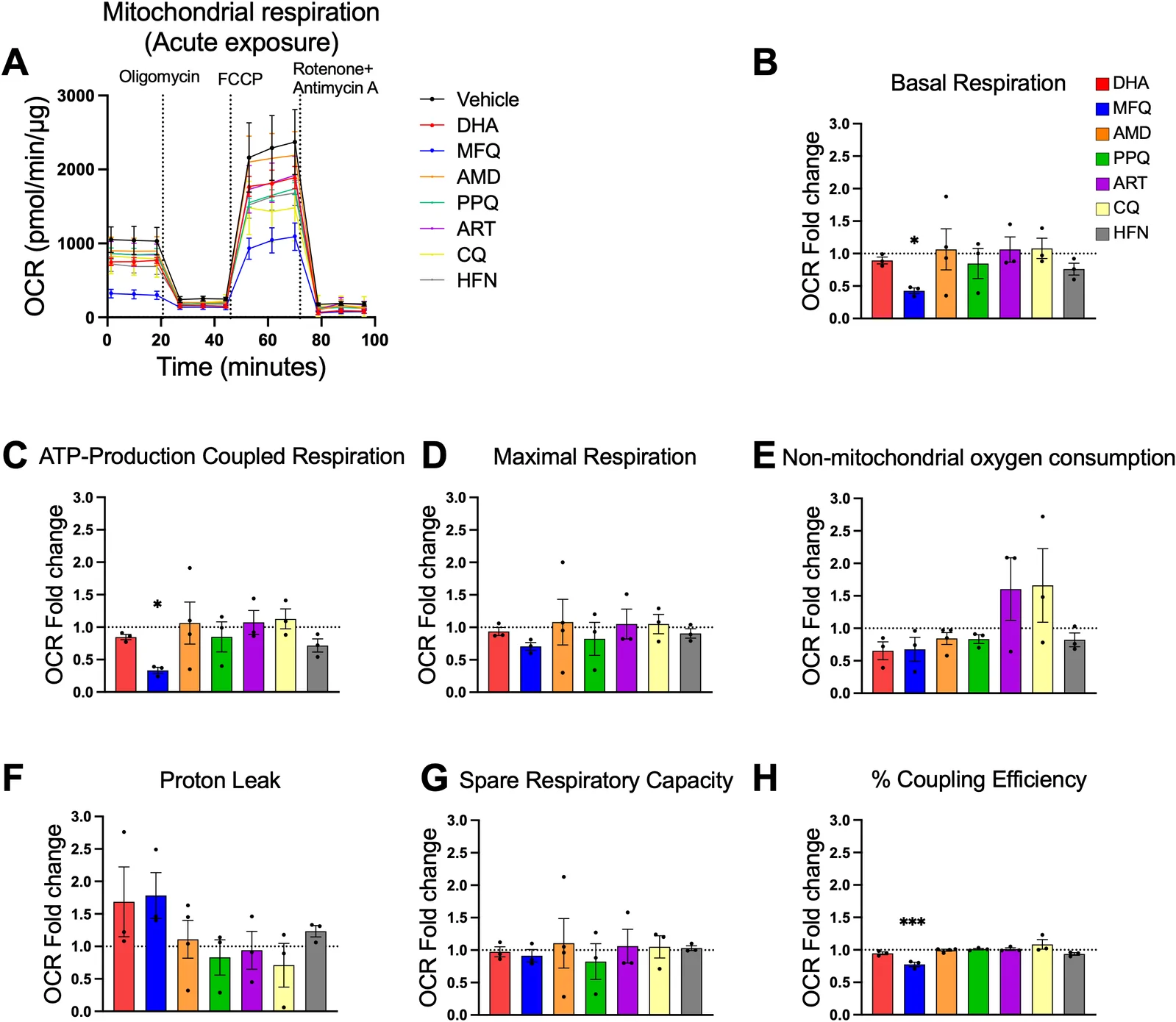
Acute effects of anti-malarial drugs on mitochondrial respiration in atrial cardiomyocytes. A) Representative respiratory profile and (B–H) quantified parameters of mitochondrial function in human atrial iPSCs after 90 minutes of single anti-malarial drug treatment. Parameters measured using the Seahorse Mito Stress Test include (B) basal respiration, (C) ATP production-coupled respiration, (D) maximal respiration, (E) non-mitochondrial oxygen consumption, (F) proton leak, (G) spare respiratory capacity, and (H) coupling efficiency (%). Results are presented as fold-change relative to control cells for each cell line. The horizontal dashed line at y = 1 indicates the oxygen consumption rate under control conditions. Data are presented as mean ±SEM, where each data point represents an individual biological replicate (n = 3 for all groups except AMD (n = 4), for which an additional replicate was included to better account for variability). Asterisks denote statistical significance of stress conditions relative to the vehicle control (one-way ANOVA): *p < 0.05; ***p < 0.001.

Based on previous studies reporting the adverse effects of anti-malarial drugs on mitochondrial respiration, we investigated the impact of acute exposure to single-dose anti-malarial drugs on the mitochondrial respiration profile of atrial cardiomyocytes. The cells were treated with DHA, MFQ, AMD, PPQ, ART, CQ, or HFN for 90 minutes (Fig 2).

Consistent with a previous published finding [5], MFQ treatment significantly reduced basal respiration, ATP-production-coupled respiration, and coupling efficiency in atrial cardiomyocytes compared to the vehicle (Fig 2). No significant alterations in mitochondrial respiration profiles were observed with the other single dose drugs, indicating that, of all the drugs tested, only MFQ exerts adverse effects on mitochondrial function during acute exposure in atrial cardiomyocytes.

### Prolonged exposure to the anti-malarial drugs Dihydroartemisinin and Halofantrine reduces mitochondrial respiration in atrial cardiomyocytes

**Fig 3:**
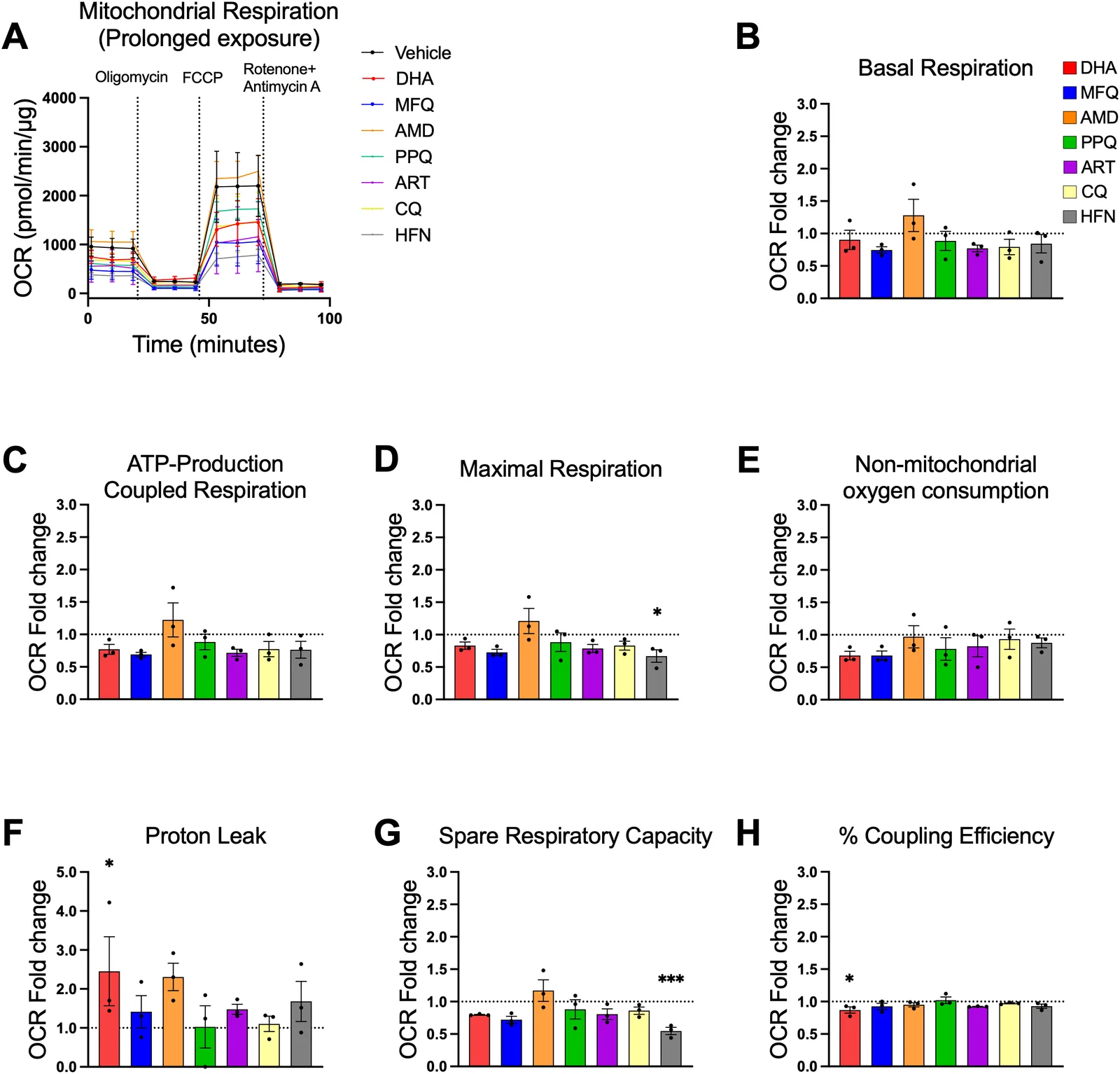
Prolonged effects of single dose anti-malarial drug treatment on mitochondrial respiration in atrial cardiomyocytes. (A) Representative respiratory profile and (B–H) quantified parameters of mitochondrial function in human atrial iPSCs after 24 hours of treatment with a single anti-malarial drug. Parameters measured using the Seahorse Mito Stress Test include (B) basal respiration, (C) ATP production-coupled respiration, (D) maximal respiration, (E) non-mitochondrial oxygen consumption, (F) proton leak, (G) spare respiratory capacity, and (H) coupling efficiency (%). Prolonged exposure to Halofantrine (HFN) significantly reduced mitochondrial respiration, particularly maximal respiration and spare respiratory capacity. Results are presented as fold-change relative to control cells for each cell line. The horizontal dashed line at y = 1 indicates the oxygen consumption rate under control conditions. Data are presented as mean ±SEM, where each data point represents an individual biological replicate (n = 3/group for all groups). Asterisks denote statistical significance of stress conditions relative to the vehicle control (one-way ANOVA): *p < 0.05; ***p < 0.001.

To investigate the long-term effects of single-dose anti-malarial drugs on mitochondrial respiration, we treated atrial cardiomyocytes with DHA, MFQ, AMD, PPQ, ART, CQ, or HFN for 24 hours and assessed mitochondrial respiration. Notably, HFN treatment significantly reduced both maximal respiration and spare respiratory capacity in atrial cardiomyocytes (Fig 3). Furthermore, DHA treatment led to reduced coupling efficiency and a significant increase in proton leak, indicating signs of mitochondrial damage in atrial cardiomyocytes.

While MFQ exhibited a trend towards reduced mitochondrial respiration, consistent with observations from acute exposure, the changes were not statistically significant. Similarly, no significant changes in mitochondrial respiration were observed in atrial cardiomyocytes treated with the remaining single-dose anti-malarial drugs for 24 hours (Fig 3), suggesting their relative safety in terms of mitochondrial function under the tested conditions.

### Acute exposure to combination anti-malarial drugs involving Mefloquine reduces mitochondrial respiration in atrial cardiomyocytes

**Fig 4:**
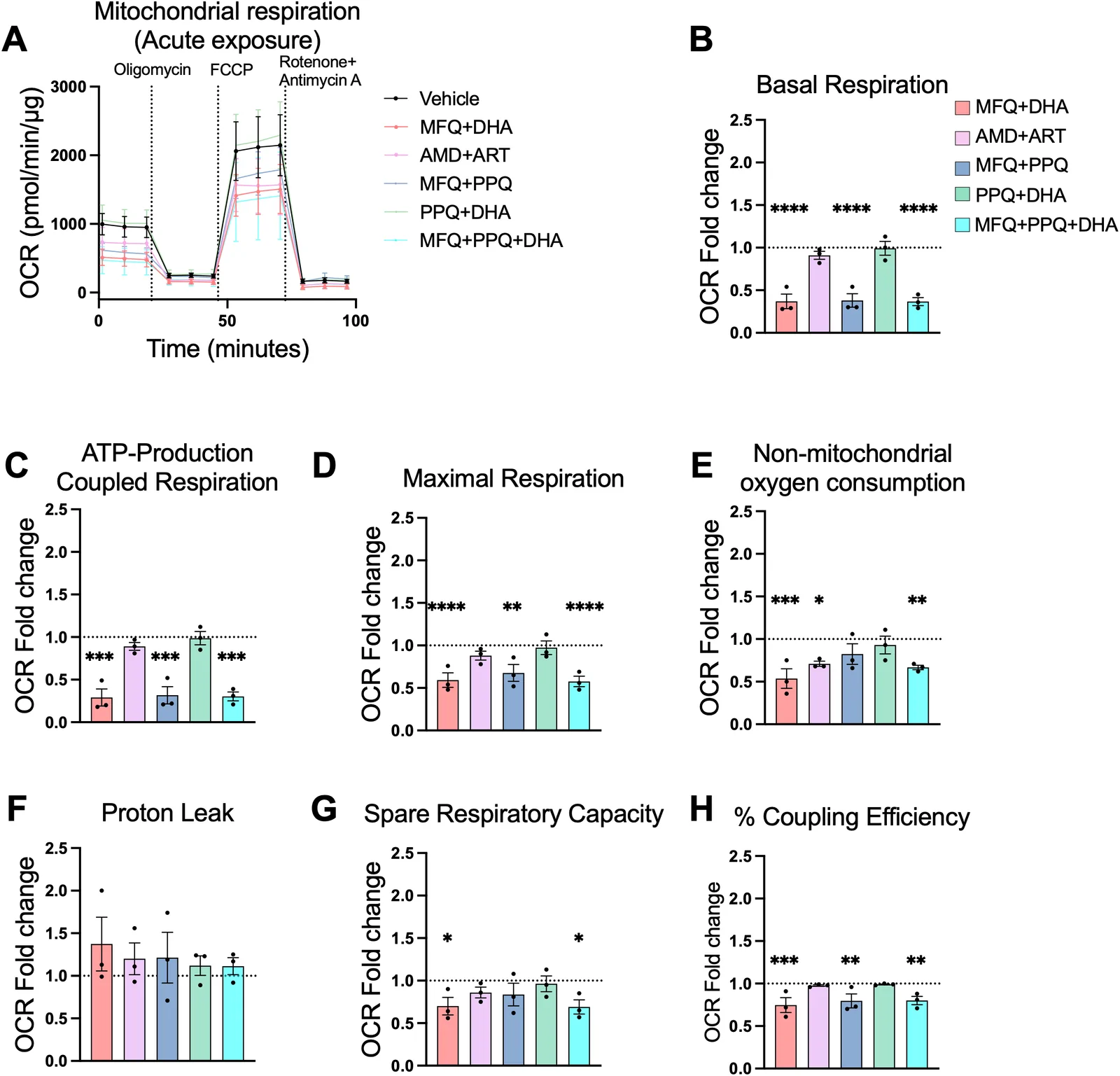
Acute exposure to combination anti-malarial treatments involving MFQ reduced mitochondrial respiration in atrial cardiomyocytes. (A) Representative respiratory profile and (B–H) quantified parameters of mitochondrial function in human atrial iPSCs after 90 minutes of treatment with a combination of two or three anti-malarial drugs. Parameters measured using the Seahorse Mito Stress Test include (B) basal respiration, (C) ATP production-coupled respiration, (D) maximal respiration, (E) non-mitochondrial oxygen consumption, (F) proton leak, (G) spare respiratory capacity, and (H) coupling efficiency (%). Results are presented as fold-change relative to control cells for each cell line. The horizontal dashed line at y = 1 indicates the oxygen consumption rate under control conditions. Drug combinations involving MFQ (mefloquine) were associated with a significant reduction in mitochondrial respiration profile. Data are presented as mean ±SEM, where each data point represents an individual biological replicate (n = 3/group for all groups). Asterisks denote statistical significance of stress conditions relative to the vehicle control (one-way ANOVA): *p < 0.05; **p < 0.01; ***p < 0.001; ****p < 0.0001.

Next, we investigated the acute effects of combination anti-malarial drug treatments on atrial cardiomyocytes. Atrial cardiomyocytes were treated with the following drug combinations: MFQ + DHA, AMD + ART, MFQ + PPQ, PPQ + DHA, and MFQ + PPQ + DHA. All combinations involving MFQ resulted in significantly reduced mitochondrial respiration, including basal respiration, ATP-production coupled respiration, maximal respiration, non-mitochondrial oxygen consumption, spare respiratory capacity, and coupling efficiency (Fig 4). No significant changes in the mitochondrial respiration were observed with combination drugs AMD + ART, and PPQ + DHA. These findings suggest that acute exposure to MFQ-containing drug combinations impair mitochondrial function in atrial cardiomyocytes.

### Prolonged exposure to combination anti-malarial drugs reduces mitochondrial respiration in atrial cardiomyocytes

**Fig 5:**
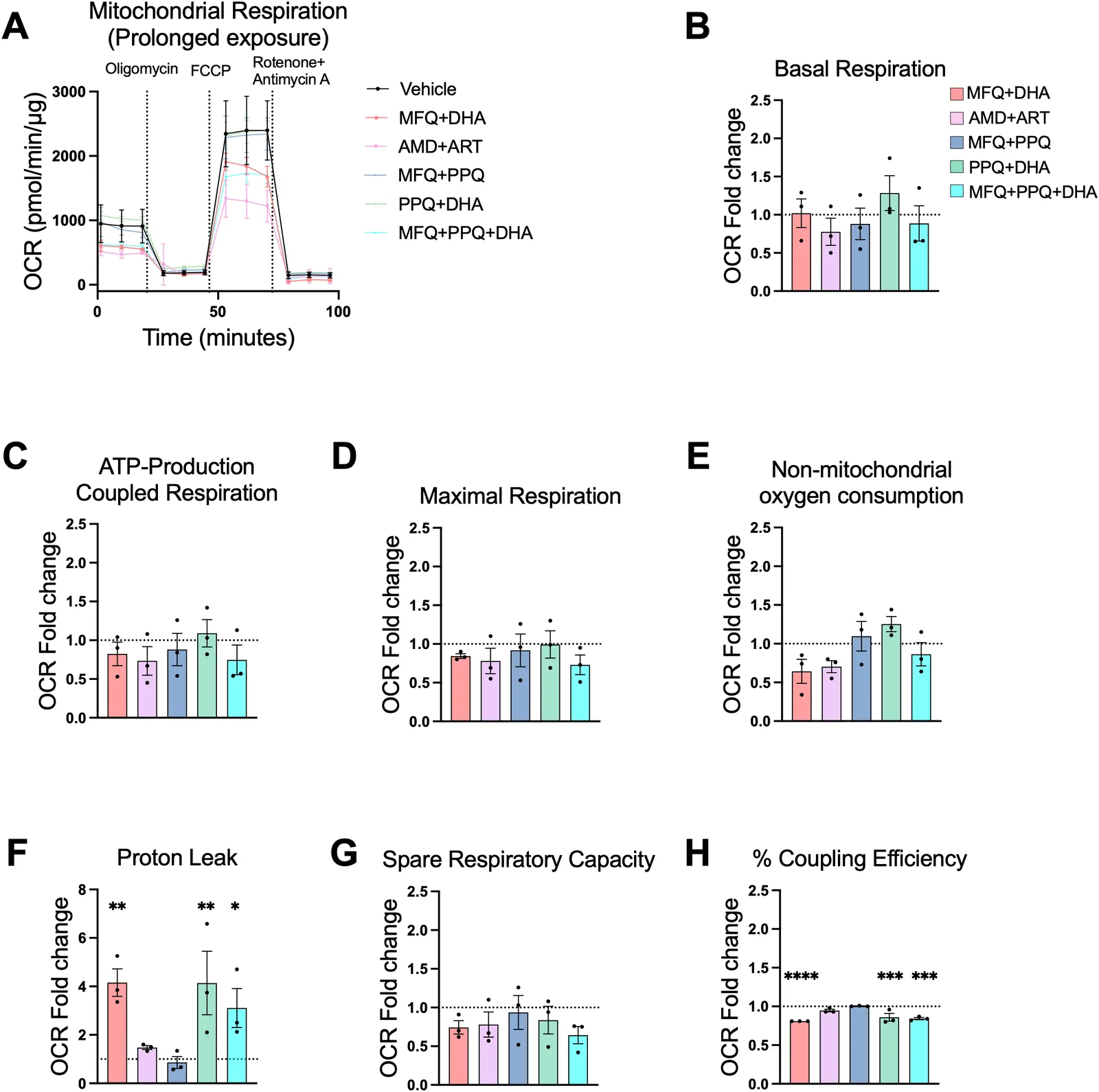
Prolonged effects of combination anti-malarial drug treatments on mitochondrial respiration in atrial cardiomyocytes. (A) Representative respiratory profile and (B–H) quantified parameters of mitochondrial function in human atrial iPSCs after 24 hours of treatment with a combination of two or three anti-malarial drugs. Parameters measured using the Seahorse Mito Stress Test include (B) basal respiration, (C) ATP production-coupled respiration, (D) maximal respiration, (E) non-mitochondrial oxygen consumption, (F) proton leak, (G) spare respiratory capacity, and (H) coupling efficiency (%). Results are presented as fold-change relative to control cells for each cell line. The horizontal dashed line at y = 1 indicates the oxygen consumption rate under control conditions. Data are presented as mean ±SEM, where each data point represents an individual biological replicate (n = 3/group for all groups). Asterisks denote statistical significance of stress conditions relative to the vehicle control (one-way ANOVA): *p < 0.05; **p < 0.01; ***p < 0.001; ****p < 0.0001.

We further investigated the prolonged effects of combination anti-malarial drugs on mitochondrial respiration in atrial cardiomyocytes, using the same drug combinations as previously described. Combination treatments involving MFQ (MFQ + DHA and MFQ + PPQ + DHA) resulted in significantly reduced coupling efficiency, indicating a decrease in the number of ATP molecules produced per O_2_ consumed (Fig 5). These treatments also caused an increase in proton leak, suggesting potential mitochondrial damage.

Contrary to the acute response, no significant changes were observed in other mitochondrial respiration parameters with MFQ-containing combination drugs, despite a trend toward reduction (Fig 5). Notably, the PPQ + DHA combination treatment also led to reduced coupling efficiency and increased proton leak, further indicating signs of mitochondrial dysfunction.

### Acute exposure to Ivermectin does not affect mitochondrial respiration in atrial cardiomyocytes, while prolonged exposure impairs coupling efficiency

Ivermectin (IVM) is a broad‐spectrum, anti‐parasitic drug widely used in the treatment of several parasitic diseases in animals and humans [28]. In humans, IVM is particularly effective in treating onchocerciasis (commonly known as River Blindness) and lymphatic filariasis [29,30], often administered through mass treatment campaigns [31]. Additionally, IVM administration has been shown to reduce malaria parasite transmission [32,33]. Notably, multiple malaria vector species have demonstrated susceptibility to IVM concentrations achievable through oral or injectable treatment at recommended doses, which are safe and effective for eliminating internal parasites [33].

**Fig 6:**
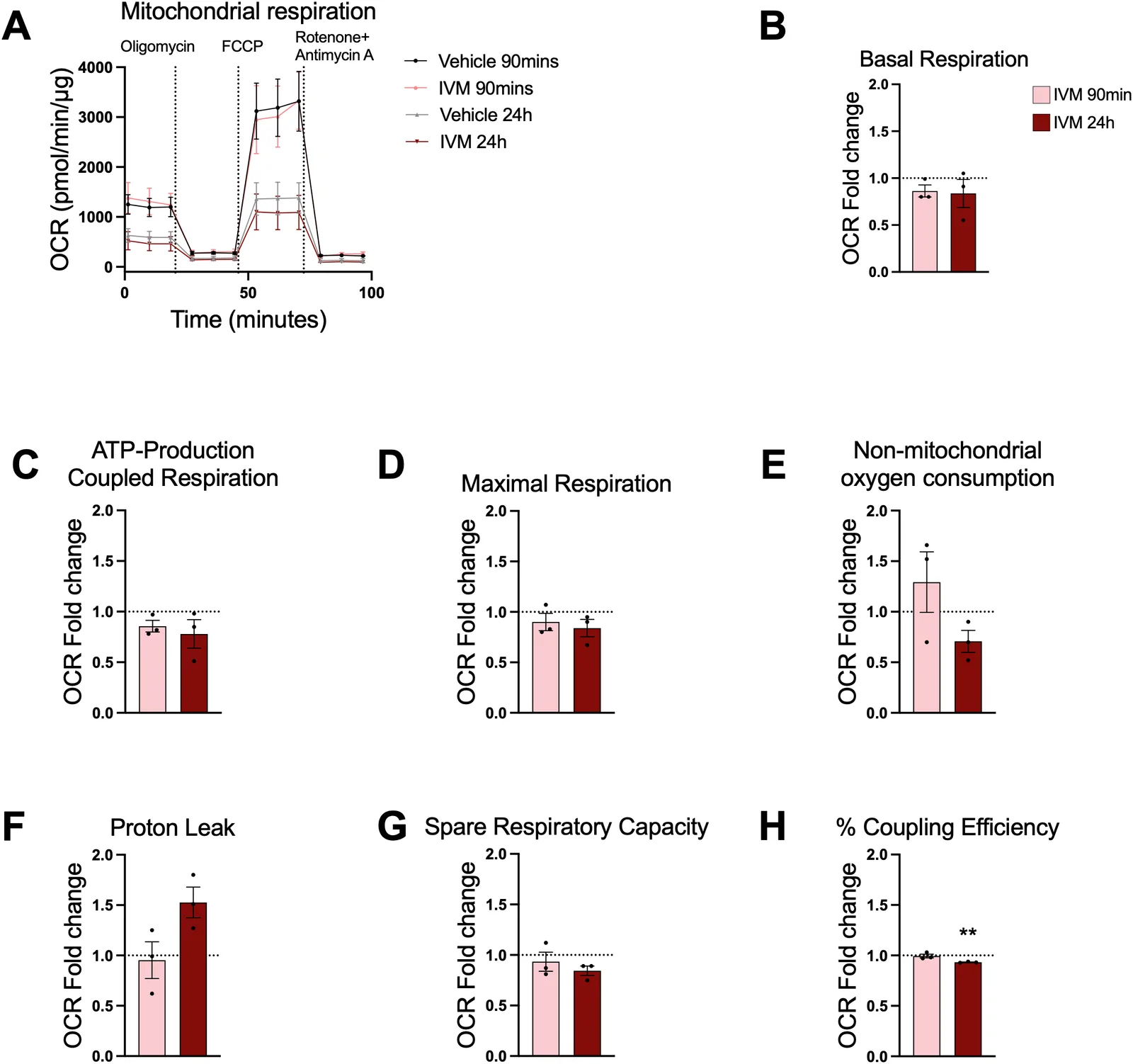
Acute exposure to Ivermectin does not affect mitochondrial respiration in atrial cardiomyocytes, while prolonged exposure reduces mitochondrial coupling efficiency. (A) Representative respiratory profile and (B–H) quantified parameters of mitochondrial function in human atrial iPSCs after 90 minutes or 24 hours treatment with Ivermectin. Parameters measured using the Seahorse Mito Stress Test include (B) basal respiration, (C) ATP production-coupled respiration, (D) maximal respiration, (E) non-mitochondrial oxygen consumption, (F) proton leak, (G) spare respiratory capacity, and (H) coupling efficiency (%). Results are presented as fold-change relative to control cells for each cell line. The horizontal dashed line at y = 1 indicates the oxygen consumption rate under control conditions. Data are presented as mean ±SEM, where each data point represents an individual biological replicate (n = 3/group for all groups). Statistical significance between treatment versus vehicle determined by Welch’s t-test: **p < 0.01.

Given these findings, we evaluated the acute effect of IVM on mitochondrial respiration in atrial cardiomyocytes. Atrial cardiomyocytes were treated with IVM for 90 minutes or 24 hours, and their mitochondrial respiration profile was assessed using the extracellular flux analyser. The results showed that acute exposure to IVM produced a mitochondrial respiration profile comparable to that of the vehicle in atrial cardiomyocytes (Fig 6). Meanwhile, prolonged exposure to IVM significantly reduced coupling efficiency without altering basal respiration, maximal respiration, spare respiratory capacity, or other mitochondrial respiration parameters (Fig 6). This finding suggests that mitochondrial energy production becomes less efficient, with fewer ATP molecules produced per unit of oxygen consumed with prolonged exposure to IVM.

The reduction in coupling efficiency may indicate increased proton leak, which is observed as a modest increase following IVM treatment (Fig 6F), or inefficiencies in the oxidative phosphorylation process, potentially signalling early mitochondrial stress. However, the absence of significant alterations in other mitochondrial parameters suggests that prolonged exposure to IVM does not induce widespread mitochondrial dysfunction under the tested conditions. These results imply that IVM may have a localised impact on mitochondrial efficiency without compromising the overall respiratory capacity of the mitochondria in atrial cardiomyocytes. Further studies are required to determine whether the observed changes in coupling efficiency have long-term functional implications for cardiomyocyte health and energy metabolism.

## Discussion

Anti-malarial drugs primarily target the asexual blood stage of *Plasmodium falciparum*, which is responsible for the majority of morbidity and mortality among patients [9]. Although the sexual stage of the parasite, gametocytes, does not cause disease directly, it plays a critical role in transmission to mosquitoes, which then infect humans. Resistance to multiple anti-malarial drugs, particularly *P. falciparum*, has emerged in many sub-Saharan regions, leading to the widespread use of combination therapies. Artemisinin and its derivatives, including artemether, are first-line treatments known for their rapid onset and wide distribution [34]. Once metabolised into DHA in the body, these drugs act quickly to eliminate the parasite and reduce fever, while also inhibiting the development of severe malaria and delaying drug resistance [9]. Although ACTs are effective at rapidly reducing gametocyte levels in the body, they do not significantly improve the prevention of infection recurrence or overall treatment success [9]. Additionally, combination therapies enhance the absorption of artemisinin and its derivatives, reduce gametocyte load, and further help delay resistance development [8].

As part of the malaria elimination efforts, mass drug administration (MDA) has been introduced [35]. However, MDA faces limitations, including concerns about the efficacy, sustainability, and operational feasibility of the drugs, unclear objectives for MDA programs, and issues related to drug resistance [35]. Therefore, assessing the safety of anti-malarial drugs prior to widespread administration is crucial. One significant challenge associated with anti-malarial drugs is their potential cardiotoxicity, particularly the risk of arrhythmias [3,4]. This risk is suggested to arise from action potential abnormalities, which may result from effects on sodium or potassium currents, calcium homeostasis, mitochondrial function, ROS production, and cardiac fibrosis [3]. Mitochondria play a key role in maintaining intracellular ion homeostasis, particularly of Na ⁺ , K ⁺ , and Ca² ⁺ , thereby preserving normal myocardial contractility and preventing electrophysiological disturbances that can lead to cardiac hypertrophy and arrhythmias [16]. In this study, we examine how single and combination anti-malarial treatments affect mitochondrial function, particularly mitochondrial respiration, during acute and prolonged exposure in atrial cardiomyocytes. Assessing OCR parameters using a Seahorse flux analyser provides functional insight into mitochondrial respiration, including ATP-linked respiration, proton leak, and spare respiratory capacity. Impairments in these parameters indicate reduced energetic capacity and increased oxidative stress, which can disrupt ion homeostasis and promote electrophysiological instability, thereby increasing the risk of arrhythmias and other adverse cardiac outcomes. Reduced ATP availability also compromises myocardial contraction and calcium handling, leading to decreased stroke volume and, consequently, reduced cardiac output.

Among single-dose drugs tested for acute effects, MFQ was the only drug to significantly impair mitochondrial function in atrial cardiomyocytes. Following a 90-minute MFQ treatment, atrial cardiomyocytes exhibited reduced basal respiration, ATP-production coupled respiration, and coupling efficiency compared to the vehicle. These findings indicate a reduced capacity for ATP production, potentially impairing oxygen utilisation efficiency to sustain cellular performance [36]. This aligns with previous studies demonstrating that MFQ inhibits mitochondrial function by suppressing mitochondrial respiration, reducing membrane potential, increasing ROS generation, and lowering ATP levels in HeLa cells [5]. Consistent with the findings in HeLa cell, we observed that MFQ includes mitochondrial stress in the human iPSC cardiomyocytes. It was unclear that whether MFQ induces clinically relevant arrhythmias. Although MFQ inhibits hERG potassium channel in transfected HEK293 cell line [37], there is no convincing clinical evidence that MFQ causes arrhythmias [38]. Our findings suggest the potential cardiotoxicity of MFQ. However, the prolonged exposure to MFQ did not significantly alter mitochondrial respiration in atrial cardiomyocytes, possibly due to a reduction in the drug’s half-life or activation of compensatory mechanisms that requires further investigation.

In contrast, prolonged exposure to HFN significantly reduced the spare respiratory capacity and coupling efficiency, indicating impaired ATP-yielding capacity. HFN has been known to inhibit *I*_Kr_ potassium channel via ROS-induced degeneration of potassium channel proteins, potentially leading to QT prolongation in human iPSC cardiomyocyte [37]. The increased mitochondrial stress identified in this study constitutes an underlying ROS-generating mechanisms that contributes to *I*_Kr_ channel reduction, thereby providing theoretical support for earlier findings. Importantly, these findings are consistent with reported adverse effects of HFN administration in patients, including prolongation of the electrocardiographic QT interval and an increased risk of sudden death [4,39,40].

Similarly, 24-hour DHA treatment resulted in decreased coupling efficiency and increased proton leak, suggesting potential mitochondrial dysfunction. These findings highlight the differential effects of anti-malarial drugs on mitochondrial function, with acute MFQ exposure exerting the most pronounced impact, while prolonged exposure to HFN and DHA reveals distinct patterns of mitochondrial impairment. Future studies will help elucidate the mechanisms underlying these alterations in mitochondrial respiration and assess the long-term safety following the use of these anti-malarial drugs.

Malaria is a global public health problem responsible for a significant number of deaths worldwide each year. One of the major limitations of monotherapy in malaria treatment is the rapid development of drug resistance. Combination therapy, using two or more drugs concurrently, provides a more effective approach to limit resistance and improve treatment durability.

We found that, similar to MFQ alone, treatment with MFQ + DHA, MFQ + PPQ, and MFQ + PPQ + DHA acutely reduced overall mitochondrial respiration in cardiomyocytes, indicating mitochondrial dysfunction, where cells are unable to generate sufficient ATP to sustain their function. Interestingly, prolonged exposure to these combination drugs did not significantly affect most of mitochondrial respiration parameters, only reducing coupling efficiency and increasing proton leak, which could be due to the reasons discussed above. Notably, prolonged exposure to PPQ + DHA also resulted in reduced coupling efficiency and increased proton leak, suggesting potential mitochondrial damage.

As an alternative to conventional anti-malarial drugs, the anti-parasitic drug ivermectin (IVM) has been proposed as a potential treatment option for malaria [32,33]. IVM is widely used to treat parasitic diseases such as lymphatic filariasis and river blindness, and it has an additional benefit of killing mosquitoes that feed on the blood of individuals who have taken the medication [29,32]. In Tanzania, the combination of albendazole and ivermectin has been adopted as part of a community-based strategy for the elimination of lymphatic filariasis, with studies suggesting that this combination is safe and effective, even in areas co-endemic with onchocerciasis [41]. However, studies conducted on five-year-old children who received ivermectin did not demonstrate significant differences in malaria prevalence between the treatment and control groups [32]. Nevertheless, it is believed that ivermectin may be more effective if administered to an entire community, where it could potentially reduce malaria transmission.

Several studies suggest a role of IVM in mitochondrial ATP production [42,43,]. For instance, Nagai et al. reported that IVM increased mitochondrial ATP production by inducing heart/muscle-specific cytochrome C oxidase subunit 6a2 (Cox6a2), a key component of the mitochondrial respiratory chain, in human iPSC-derived cardiomyocytes [42]. Conversely, other reports have highlighted the apoptotic effects of IVM, particularly in chronic myeloid leukaemia, by inducing mitochondrial dysfunction and oxidative stress, potentially through inhibition of the Akt/mTOR pathway [43]. This reported paradoxical mitochondrial modulation underscores the complex and context-dependent nature of IVM’s effects on mitochondrial function. In this study we show that acute exposure to IVM did not adversely affect mitochondrial respiration. However, prolonged exposure to IVM led to a reduction in coupling efficiency, while other mitochondrial respiration parameters remained unaffected. These findings suggest that IVM has the potential to cause subtle changes in mitochondrial efficiency, which requires further investigation to fully elucidate its long-term effects on cardiomyocyte function.

## Conclusion

This study provides valuable insights into the effects of anti-malarial drugs on mitochondrial function in atrial cardiomyocytes. The findings demonstrate that while certain drugs, such as MFQ and its combinations, have acute detrimental effects on mitochondrial respiration, other drugs including DHA, HFN, or IVM appear to have no significant acute impact but may cause subtle long-term changes. This comprehensive understanding of drug effects can guide safer and more effective treatment strategies for malaria. Additional mechanistic studies could enhance our knowledge of the broader cellular implications of these drugs, supporting the development of improved therapeutic approaches.

## Abbreviations

LMICs: Low- and middle-income countries
ROS: Reactive oxygen species
WHO: World Health Organization
ACTs: Artemisinin-based combination therapies
BPM: Beats per minute
Ca^2+^: Calcium
DHA: Dihydroartemisinin
MFQ: Mefloquine hydrochloride
ART: Artemether
IVM: Ivermectin
CQ: Chloroquine phosphate
HFN: Halofantrine Hydrochloride
AMD: Amodiaquine Hydrochloride Hydrate
PPQ: Piperaquine
iPSC: induced pluripotent stem cells
OCR: Oxygen consumption rate
ATP: Adenosine triphosphate
FCCP: Carbonyl cyanide-p-trifluoromethoxyphenylhydrazone
SEM: Standard error of the mean
