# Supplementary material for: Acute and prolonged effects of anti-malarial drugs on mitochondrial respiration in atrial cardiomyocytes for cardiac safety evaluation
Source: PLoS One. 2026 Sep 11;21(9):e0351511. doi: 10.1371/journal.pone.0351511 (PMC13567763; doi:10.1371/journal.pone.0351511)
Supplement: Supplementary file 1 [file pone.0351511.s002.docx]

**Response to Reviewers**

Dear Editor and Reviewer,

Thank you for your time and constructive feedback on our manuscript. We have revised it accordingly and are pleased to submit an improved version, which we hope meets your satisfaction.

Yours sincerely,

Kamalan Jeevaratnam on behalf of all co-authors

**PONE-D-26-11390**

**Acute and Prolonged Effects of Anti-Malarial Drugs on Mitochondrial Respiration in Atrial Cardiomyocytes for Cardiac Safety Evaluation**

| **Editorial:** |  |
| --- | --- |
| Please ensure that your manuscript meets PLOS ONE's style requirements, including those for file naming. | Checked. |
| Edit financial disclosure | The financial disclosure is added in the submission system.  “This project was funded by the British Heart Foundation PG/22/10899 awarded to Kamalan Jeevaratnam. The funders had no role in study design, data collection and analysis, decision to publish, or preparation of the manuscript." |
| Edit Acknowledgments Section | This section has been removed from the manuscript. |
| Submit to a repository upon acceptance | Yes, will do. |
| review your reference list | Checked and edited as needed. |
|  |  |
| **Reviewer:** | **NB. Lines given is with “simple markup”** |
| A. Add 2–3 sentences in Introduction + Discussion: |  |
| 1. Explain how mitochondrial dysfunction → electrophysiological instability → arrhythmia risk | **For introduction lines 102-110 added:** Given the high energy demands of cardiomyocytes, mitochondrial dysfunction has profound effects on cellular electrophysiology. Mitochondria play a critical role in myocardial contraction by regulating intracellular Na+, K+, and Ca2+ levels [15], [16]. Reduced ATP production impairs the activity of ion pumps such as the Na⁺/K⁺-ATPase and Ca²⁺-ATPase, leading to ionic imbalance, while increased ROS levels can modify ion channel function. Furthermore, disrupted mitochondrial calcium handling promotes intracellular Ca²⁺ overload, which can trigger delayed afterdepolarisations [16], [17]. Together, these alterations contribute to electrophysiological instability, thereby increasing the risk of cardiac arrhythmias and arrhythmia-related sudden cardiac death.  **For discussion lines 281-284:** Mitochondria play a key role in maintaining intracellular ion homeostasis, particularly of Na⁺, K⁺, and Ca²⁺, thereby preserving normal myocardial contractility and preventing electrophysiological disturbances that can lead to cardiac hypertrophy and arrhythmias. |
| 2. Link OCR parameters to real cardiac outcomes | Thank you for this suggestion. We have expanded the discussion to include:  **Lines 286-293:** Assessing OCR parameters using a Seahorse flux analyser provides functional insight into mitochondrial respiration, including ATP-linked respiration, proton leak, and spare respiratory capacity. Impairments in these parameters indicate reduced energetic capacity and increased oxidative stress, which can disrupt ion homeostasis and promote electrophysiological instability, thereby increasing the risk of arrhythmias and other adverse cardiac outcomes. Reduced ATP availability also compromises myocardial contraction and calcium handling, leading to decreased stroke volume and, consequently, reduced cardiac output. |
| B. **Experimental Design, please add** |  |
| 1. Citation for each drug concentration | We apologise for this omission. Now added, see below. |
| 2. Clarify: Peak plasma (Cmax)?, Free drug concentration?, Tissue vs plasma relevance? | Edited text under subheading **Drug Treatments** (line 126-140).  “The in vitro drug concentrations used in this study were determined based on the therapeutic concentrations employed clinically, with reference to peak plasma (Cmax) data, and were applied according to total plasma concentrations. For combination therapies, the experiments were conducted using concentrations corresponding to those used in the fixed-dose formulations, and the experimental concentrations for the single agents were equal to those used in the combination therapies. The pharmacological data for each individual drug were referred to the following sources: dihydroartemisinin (DHA, 1.75 μM, [18], Cayman Chemical, USA) and piperaquine (PPQ, 0.5 μM, [19], Cayman Chemical, USA), both dissolved in DMSO. Chloroquine phosphate (CQ, 3 μM, [20], Sigma Aldrich, UK) was dissolved in ddH₂O, while the following drugs were dissolved in methanol: artemether (ART, 0.7 μM, [21], Sigma Aldrich, UK), amodiaquine hydrochloride (AMD, 0.03 μM, [22], Sigma Aldrich, UK), halofantrine hydrochloride (HFN, 2 μM, [23], Sigma Aldrich, UK), mefloquine hydrochloride (MFQ, 4 μM, [24], Sigma Aldrich, UK), and ivermectin (IVM, 0.1 μM, [25], Sigma Aldrich, UK). Vehicle solutions were matched to the diluents and concentrations used for the drug treatments. |
| 3. Explain: 90 min = acute pharmacological effect, and 24 h = accumulation / mitochondrial adaptation | This has been further explained in the text.  **Line 142-145:** The acute exposure was designed based on the minimum exposure time recommended by the manufacturer, while the prolonged exposure condition was set as 24 hours, referring to a previous study showing that mitochondrial respiration parameters such as proton leak and coupling efficiency undergo significant remodelling following 24 hours cellular stress exposure in cardiomyoblasts [26]. Moreover, 24 hours exposure duration is commonly used to evaluate rapid parasite killing kinetics and stage-specific antimalarial activity in Plasmodium falciparum in vitro assays [27]. |
| C. **Statistical** |  |
| 1. Clarify: What counts as “biological replicate”? (different passages? batches?) statistically. | **Lines 171-172 were updated in methods section under statistical analysis:**  All experiments included three biological replicates from independent manufacturer batches. Statistical significance was defined as p<0.05. |
| 2. Add: Exact n per condition in figure legends, Effect sizes (not just p-values) | Biological n values are already stated in every figure legend “Data are presented as mean ±SEM, with n=3/group”. A small correction on our end in Figure 1 legends for n number which is now corrected to: “Data are presented as mean ±SEM, with n=3 for all groups except AMD (n = 4), for which an additional replicate was included to better account for variability.” |
| 3. If possible, increase n or justify power statistically. | We thank the reviewer for this valid suggestion. To justify the sample size, we have done post hoc ANOVA-based power analyses on representative Seahorse parameters (basal respiration and coupling efficiency) across both timepoints and treatment conditions.  Most analyses showed adequate to high statistical power, particularly for coupling efficiency measurements (power = 0.93–1.00), while basal respiration datasets showed moderate to high power depending on the experimental condition (power = 0.58-1.00). These analyses support that the study was sufficiently powered to detect biologically relevant changes in mitochondrial function.  Text added into material and methods, statistical analysis, **line 173-176**:  “Post hoc ANOVA-based power analyses were performed on representative Seahorse parameters, including basal respiration and coupling efficiency, to confirm adequate statistical sensitivity for detecting treatment-associated effects.” |
| D. **Interpretation** |  |
| 1. You conclude: “relatively safe in terms of mitochondrial function”. This is too strong. Suggested rewrite, Replace with: “did not significantly alter mitochondrial respiration under the tested conditions” | “highlighting their relative safety in terms of mitochondrial function.”  **Line 203-204**, changed to:  “Suggesting their relative safety in terms of mitochondrial function under the tested conditions.” |
| E. **Suggested additions (Discussion) For key findings:** |  |
| 1. MFQ, Mention: mitochondrial membrane potential disruption, ROS generation, ETC inhibition | Thank you for your suggestion. We have revised the manuscript to further elaborate on our findings.  (**Line 304**) “Consistent with the findings in HeLa cell, we observed that MFQ incudes mitochondrial stress in the human iPSC cardiomyocytes. It was unclear whether MFQ induces clinically relevant arrhythmias. Although MFQ inhibits hERG potassium channel in transfected HEK293 cell line [37], there is no convincing clinical evidence that MFQ causes arrhythmias [38]. Our findings suggest the potential cardiotoxicity of MFQ.” |
| 2. HFN, Link to: QT prolongation, ion channel effects + mitochondrial stress | (**Line 313-320**). HFN has been known to inhibit *I*_Kr_ potassium channel via ROS-induced degeneration of potassium channel proteins, potentially leading to QT prolongation in human iPSC cardiomyocyte [39]. The increased mitochondrial stress identified in this study constitutes an underlying ROS-generating mechanisms that contributes to *I*_Kr_ channel reduction, thereby providing theoretical support for earlier findings. Importantly, these findings are consistent with reported adverse effects of HFN administration in patients, including prolongation of the electrocardiographic QT interval and an increased risk of sudden death.” |
| 3. IVM, Discuss: paradoxical mitochondrial modulation (literature conflict) | To clarify in the discussion about ivermectin we have added a sentence to acknowledge the paradox.  **Line 359-361**: “This reported paradoxical mitochondrial modulation underscores the complex and context-dependent nature of IVM's effects on mitochondrial function. “ |
| F. **Figures (Clarity Issues), From Figures (pages 32–36)** |  |
| 1. Increase font size | Font size increased for all figures. |
| 2. Add: sample size (n) directly in figure, clear color legend consistency | The sample sizes are already stated in the figure legends, and each biological replicate is represented in the figures as dots in the columns for each condition. The dot size has been increased for better clarity. This information is also added to the figure legends for clarity. |
| 3. Consider: highlighting key comparisons (e.g., MFQ vs control) | The figure legends are updated for clarity for all figures. For example Figure 1: “The horizontal dashed line at y=1 indicates the oxygen consumption rate under control conditions. Data are presented as mean ±SEM, with n=3 for all groups except AMD (n = 4), for which an additional replicate was included to better account for variability. Asterisks denote statistical significance of stress conditions relative to the vehicle control (one-way ANOVA): *p < 0.05; ***p < 0.001. |
| G. **Vehicle control clarity** |  |
| 1. Add clarification: “No differences were observed between vehicle controls” | Indeed, the vehicle solutions showed no differences. This has been added to Material and methods **line 140**. |
