# Supplementary material for: Acute and prolonged effects of anti-malarial drugs on mitochondrial respiration in atrial cardiomyocytes for cardiac safety evaluation
Source: PLoS One. 2026 Sep 11;21(9):e0351511. doi: 10.1371/journal.pone.0351511 (PMC13567763; doi:10.1371/journal.pone.0351511)
Supplement: Peer review history

# Peer review: Decision Letter 0

**DOI:** 10.1371/journal.pone.0351511.r001

**Authors:** José Luiz Vieira

8 Apr 2026

ACADEMIC EDITOR:

Please submit your revised manuscript by May 23 2026 11:59PM If you will need more time than this to complete your revisions, please reply to this message or contact the journal office at plosone@plos.org. When you're ready to submit your revision, log on to https://www.editorialmanager.com/pone/ and select the 'Submissions Needing Revision' folder to locate your manuscript file.

- A letter that responds to each point raised by the academic editor and reviewer(s). You should upload this letter as a separate file labeled 'Response to Reviewers'.
- A marked-up copy of your manuscript that highlights changes made to the original version. You should upload this as a separate file labeled 'Revised Manuscript with Track Changes'.
- An unmarked version of your revised paper without tracked changes. You should upload this as a separate file labeled 'Manuscript'.

If you would like to make changes to your financial disclosure, please include your updated statement in your cover letter. Guidelines for resubmitting your figure files are available below the reviewer comments at the end of this letter.

If applicable, we recommend that you deposit your laboratory protocols in protocols.io to enhance the reproducibility of your results. Protocols.io assigns your protocol its own identifier (DOI) so that it can be cited independently in the future. For instructions see: https://journals.plos.org/plosone/s/submission-guidelines#loc-laboratory-protocols. Additionally, PLOS ONE offers an option for publishing peer-reviewed Lab Protocol articles, which describe protocols hosted on protocols.io. Read more information on sharing protocols at https://plos.org/protocols?utm_medium=editorial-email&utm_source=authorletters&utm_campaign=protocols.

As the corresponding author, your ORCID iD is verified in the submission system and will appear in the published article. PLOS supports the use of ORCID, and we encourage all coauthors to register for an ORCID iD and use it as well. Please encourage your coauthors to verify their ORCID iD within the submission system before final acceptance, as unverified ORCID iDs will not appear in the published article. *Only*  the individual author can complete the verification step; PLOS staff *cannot*  verify ORCID iDs on behalf of authors.

We look forward to receiving your revised manuscript.

Kind regards,

José Luiz Fernandes Vieira

Academic Editor

PLOS One

Journal Requirements:

When submitting your revision, we need you to address these additional requirements.

1. Please ensure that your manuscript meets PLOS ONE's style requirements, including those for file naming. The PLOS ONE style templates can be found at

https://journals.plos.org/plosone/s/file?id=wjVg/PLOSOne_formatting_sample_main_body.pdf and

https://journals.plos.org/plosone/s/file?id=ba62/PLOSOne_formatting_sample_title_authors_affiliations.pdf

2. Thank you for stating the following financial disclosure: [This project is funded by the British Heart Foundation PG/22/10899 award to Kamalan Jeevaratnam].

Please state what role the funders took in the study.  If the funders had no role, please state: ""The funders had no role in study design, data collection and analysis, decision to publish, or preparation of the manuscript.""

If this statement is not correct you must amend it as needed.

Please include this amended Role of Funder statement in your cover letter; we will change the online submission form on your behalf.

3. Thank you for stating the following in the Acknowledgments Section of your manuscript: [This project is funded by the British Heart Foundation PG/22/10899 award to Kamalan Jeevaratnam]

We note that you have provided funding information that is not currently declared in your Funding Statement. However, funding information should not appear in the Acknowledgments section or other areas of your manuscript. We will only publish funding information present in the Funding Statement section of the online submission form.

Please remove any funding-related text from the manuscript and let us know how you would like to update your Funding Statement. Currently, your Funding Statement reads as follows: [This project is funded by the British Heart Foundation PG/22/10899 award to Kamalan Jeevaratnam].

Please include your amended statements within your cover letter; we will change the online submission form on your behalf.

4. In the online submission form, you indicated that your data will be submitted to a repository upon acceptance. We strongly recommend all authors deposit their data before acceptance, as the process can be lengthy and hold up publication timelines. Please note that, though access restrictions are acceptable now, your entire minimal dataset will need to be made freely accessible if your manuscript is accepted for publication. This policy applies to all data except where public deposition would breach compliance with the protocol approved by your research ethics board. If you are unable to adhere to our open data policy, please kindly revise your statement to explain your reasoning and we will seek the editor's input on an exemption.

5. If the reviewer comments include a recommendation to cite specific previously published works, please review and evaluate these publications to determine whether they are relevant and should be cited. There is no requirement to cite these works unless the editor has indicated otherwise.

6. Please review your reference list to ensure that it is complete and correct. If you have cited papers that have been retracted, please include the rationale for doing so in the manuscript text, or remove these references and replace them with relevant current references. Any changes to the reference list should be mentioned in the rebuttal letter that accompanies your revised manuscript. If you need to cite a retracted article, indicate the article’s retracted status in the References list and also include a citation and full reference for the retraction notice.

[Note: HTML markup is below. Please do not edit.]

Reviewers' comments:

Reviewer's Responses to Questions

**Comments to the Author**

1. Is the manuscript technically sound, and do the data support the conclusions?

Reviewer #1: Partly

**********

2. Has the statistical analysis been performed appropriately and rigorously?

Reviewer #1: Yes

**********

3. Have the authors made all data underlying the findings in their manuscript fully available?

The PLOS Data policy

Reviewer #1: Yes

**********

4. Is the manuscript presented in an intelligible fashion and written in standard English?

Reviewer #1: Yes

**********

Reviewer #1: Add 2–3 sentences in Introduction + Discussion:

1. Explain how mitochondrial dysfunction → electrophysiological instability → arrhythmia risk

2. Link OCR parameters to real cardiac outcomes

Experimental Design, please add

1. Citation for each drug concentration

2. Clarify: Peak plasma (Cmax)?, Free drug concentration?, Tissue vs plasma relevance?

3. Explain: 90 min = acute pharmacological effect, and 24 h = accumulation / mitochondrial adaptation

Statistical

1. Clarify: What counts as “biological replicate”? (different passages? batches?)

2. Add: Exact n per condition in figure legends, Effect sizes (not just p-values)

3. If possible, increase n or justify power statistically.

Interpretation

1. You conclude: “relatively safe in terms of mitochondrial function”. This is too strong. Suggested rewrite, Replace with: “did not significantly alter mitochondrial respiration under the tested conditions”

Suggested additions (Discussion) For key findings:

1. MFQ, Mention: mitochondrial membrane potential disruption, ROS generation, ETC inhibition

2. HFN, Link to: QT prolongation, ion channel effects + mitochondrial stress

3. IVM, Discuss: paradoxical mitochondrial modulation (literature conflict)

Figures (Clarity Issues), From Figures (pages 32–36)

1. Increase font size

2. Add: sample size (n) directly in figure, clear color legend consistency

3. Consider: highlighting key comparisons (e.g., MFQ vs control)

Vehicle control clarity

1. Add clarification: “No differences were observed between vehicle controls”

**********

what does this mean?). If published, this will include your full peer review and any attached files.

If you choose “no”, your identity will remain anonymous but your review may still be made public.

**Do you want your identity to be public for this peer review?**  For information about this choice, including consent withdrawal, please see our Privacy Policy

Reviewer #1: **Yes:** Voravuth Somsak

**********

[NOTE: If reviewer comments were submitted as an attachment file, they will be attached to this email and accessible via the submission site. Please log into your account, locate the manuscript record, and check for the action link "View Attachments". If this link does not appear, there are no attachment files.]

To ensure your figures meet our technical requirements, please review our figure guidelines: https://journals.plos.org/plosone/s/figures

You may also use PLOS’s free figure tool, NAAS, to help you prepare publication quality figures: https://journals.plos.org/plosone/s/figures#loc-tools-for-figure-preparation.

NAAS will assess whether your figures meet our technical requirements by comparing each figure against our figure specifications.

# Author comment: Author response to Decision Letter 1

**DOI:** 10.1371/journal.pone.0351511.r002

21 May 2026

Responses copied from the response letter attached.

Editorial:

Please ensure that your manuscript meets PLOS ONE's style requirements, including those for file naming.

Checked.

Edit financial disclosure

The financial disclosure is added in the submission system.

“This project was funded by the British Heart Foundation PG/22/10899 awarded to Kamalan Jeevaratnam. The funders had no role in study design, data collection and analysis, decision to publish, or preparation of the manuscript."

Edit Acknowledgments Section

This section has been removed from the manuscript.

Submit to a repository upon acceptance Yes, will do.

review your reference list Checked and edited as needed.

Reviewer: NB. Lines given is with “simple markup”

A. Add 2–3 sentences in Introduction + Discussion:

1. Explain how mitochondrial dysfunction → electrophysiological instability → arrhythmia risk For introduction lines 102-110 added: Given the high energy demands of cardiomyocytes, mitochondrial dysfunction has profound effects on cellular electrophysiology. Mitochondria play a critical role in myocardial contraction by regulating intracellular Na+, K+, and Ca2+ levels [15], [16]. Reduced ATP production impairs the activity of ion pumps such as the Na⁺/K⁺-ATPase and Ca²⁺-ATPase, leading to ionic imbalance, while increased ROS levels can modify ion channel function. Furthermore, disrupted mitochondrial calcium handling promotes intracellular Ca²⁺ overload, which can trigger delayed afterdepolarisations [16], [17]. Together, these alterations contribute to electrophysiological instability, thereby increasing the risk of cardiac arrhythmias and arrhythmia-related sudden cardiac death.

For discussion lines 281-284: Mitochondria play a key role in maintaining intracellular ion homeostasis, particularly of Na⁺, K⁺, and Ca²⁺, thereby preserving normal myocardial contractility and preventing electrophysiological disturbances that can lead to cardiac hypertrophy and arrhythmias.

2. Link OCR parameters to real cardiac outcomes Thank you for this suggestion. We have expanded the discussion to include:

Lines 286-293: Assessing OCR parameters using a Seahorse flux analyser provides functional insight into mitochondrial respiration, including ATP-linked respiration, proton leak, and spare respiratory capacity. Impairments in these parameters indicate reduced energetic capacity and increased oxidative stress, which can disrupt ion homeostasis and promote electrophysiological instability, thereby increasing the risk of arrhythmias and other adverse cardiac outcomes. Reduced ATP availability also compromises myocardial contraction and calcium handling, leading to decreased stroke volume and, consequently, reduced cardiac output.

B. Experimental Design, please add

1. Citation for each drug concentration We apologise for this omission. Now added, see below.

2. Clarify: Peak plasma (Cmax)?, Free drug concentration?, Tissue vs plasma relevance? Edited text under subheading Drug Treatments (line 126-140).

“The in vitro drug concentrations used in this study were determined based on the therapeutic concentrations employed clinically, with reference to peak plasma (Cmax) data, and were applied according to total plasma concentrations. For combination therapies, the experiments were conducted using concentrations corresponding to those used in the fixed-dose formulations, and the experimental concentrations for the single agents were equal to those used in the combination therapies. The pharmacological data for each individual drug were referred to the following sources: dihydroartemisinin (DHA, 1.75 μM, [18], Cayman Chemical, USA) and piperaquine (PPQ, 0.5 μM, [19], Cayman Chemical, USA), both dissolved in DMSO. Chloroquine phosphate (CQ, 3 μM, [20], Sigma Aldrich, UK) was dissolved in ddH₂O, while the following drugs were dissolved in methanol: artemether (ART, 0.7 μM, [21], Sigma Aldrich, UK), amodiaquine hydrochloride (AMD, 0.03 μM, [22], Sigma Aldrich, UK), halofantrine hydrochloride (HFN, 2 μM, [23], Sigma Aldrich, UK), mefloquine hydrochloride (MFQ, 4 μM, [24], Sigma Aldrich, UK), and ivermectin (IVM, 0.1 μM, [25], Sigma Aldrich, UK). Vehicle solutions were matched to the diluents and concentrations used for the drug treatments.

3. Explain: 90 min = acute pharmacological effect, and 24 h = accumulation / mitochondrial adaptation This has been further explained in the text.

Line 142-145: The acute exposure was designed based on the minimum exposure time recommended by the manufacturer, while the prolonged exposure condition was set as 24 hours, referring to a previous study showing that mitochondrial respiration parameters such as proton leak and coupling efficiency undergo significant remodelling following 24 hours cellular stress exposure in cardiomyoblasts [26]. Moreover, 24 hours exposure duration is commonly used to evaluate rapid parasite killing kinetics and stage-specific antimalarial activity in Plasmodium falciparum in vitro assays [27].

C. Statistical

1. Clarify: What counts as “biological replicate”? (different passages? batches?)

statistically.

Lines 171-172 were updated in methods section under statistical analysis:

All experiments included three biological replicates from independent manufacturer batches. Statistical significance was defined as p<0.05.

2. Add: Exact n per condition in figure legends, Effect sizes (not just p-values)

Biological n values are already stated in every figure legend “Data are presented as mean ±SEM, with n=3/group”. A small correction on our end in Figure 1 legends for n number which is now corrected to: “Data are presented as mean ±SEM, with n=3 for all groups except AMD (n = 4), for which an additional replicate was included to better account for variability.”

3. If possible, increase n or justify power statistically.

We thank the reviewer for this valid suggestion. To justify the sample size, we have done post hoc ANOVA-based power analyses on representative Seahorse parameters (basal respiration and coupling efficiency) across both timepoints and treatment conditions.

Most analyses showed adequate to high statistical power, particularly for coupling efficiency measurements (power = 0.93–1.00), while basal respiration datasets showed moderate to high power depending on the experimental condition (power = 0.58-1.00). These analyses support that the study was sufficiently powered to detect biologically relevant changes in mitochondrial function.

Text added into material and methods, statistical analysis, line 173-176:

“Post hoc ANOVA-based power analyses were performed on representative Seahorse parameters, including basal respiration and coupling efficiency, to confirm adequate statistical sensitivity for detecting treatment-associated effects.”

D. Interpretation

1. You conclude: “relatively safe in terms of mitochondrial function”. This is too strong. Suggested rewrite, Replace with: “did not significantly alter mitochondrial respiration under the tested conditions”

“highlighting their relative safety in terms of mitochondrial function.”

Line 203-204, changed to:

“Suggesting their relative safety in terms of mitochondrial function under the tested conditions.”

E. Suggested additions (Discussion) For key findings:

1. MFQ, Mention: mitochondrial membrane potential disruption, ROS generation, ETC inhibition

Thank you for your suggestion. We have revised the manuscript to further elaborate on our findings.

(Line 304) “Consistent with the findings in HeLa cell, we observed that MFQ incudes mitochondrial stress in the human iPSC cardiomyocytes. It was unclear whether MFQ induces clinically relevant arrhythmias. Although MFQ inhibits hERG potassium channel in transfected HEK293 cell line [37], there is no convincing clinical evidence that MFQ causes arrhythmias [38]. Our findings suggest the potential cardiotoxicity of MFQ.”

2. HFN, Link to: QT prolongation, ion channel effects + mitochondrial stress

(Line 313-320). HFN has been known to inhibit IKr potassium channel via ROS-induced degeneration of potassium channel proteins, potentially leading to QT prolongation in human iPSC cardiomyocyte [39]. The increased mitochondrial stress identified in this study constitutes an underlying ROS-generating mechanisms that contributes to IKr channel reduction, thereby providing theoretical support for earlier findings. Importantly, these findings are consistent with reported adverse effects of HFN administration in patients, including prolongation of the electrocardiographic QT interval and an increased risk of sudden death.”

3. IVM, Discuss: paradoxical mitochondrial modulation (literature conflict) To clarify in the discussion about ivermectin we have added a sentence to acknowledge the paradox.

Line 359-361: “This reported paradoxical mitochondrial modulation underscores the complex and context-dependent nature of IVM's effects on mitochondrial function. “

F. Figures (Clarity Issues), From Figures (pages 32–36)

1. Increase font size Font size increased for all figures.

2. Add: sample size (n) directly in figure, clear color legend consistency The sample sizes are already stated in the figure legends, and each biological replicate is represented in the figures as dots in the columns for each condition. The dot size has been increased for better clarity. This information is also added to the figure legends for clarity.

3. Consider: highlighting key comparisons (e.g., MFQ vs control) The figure legends are updated for clarity for all figures. For example Figure 1: “The horizontal dashed line at y=1 indicates the oxygen consumption rate under control conditions. Data are presented as mean ±SEM, with n=3 for all groups except AMD (n = 4), for which an additional replicate was included to better account for variability. Asterisks denote statistical significance of stress conditions relative to the vehicle control (one-way ANOVA): *p < 0.05; ***p < 0.001.

G. Vehicle control clarity

1. Add clarification: “No differences were observed between vehicle controls”

Indeed, the vehicle solutions showed no differences. This has been added to Material and methods line 140.

Attachment Submitted filename: 21.5.2026_Response to reviewers.docx

# Editor report: Decision Letter 1

**DOI:** 10.1371/journal.pone.0351511.r003

**Authors:** José Luiz Vieira

29 May 2026

<p>Acute and Prolonged Effects of Anti-Malarial Drugs on Mitochondrial Respiration in Atrial Cardiomyocytes for Cardiac Safety Evaluation

PONE-D-26-11390R1

Dear Dr.Kamalan Jeevaratnam

We’re pleased to inform you that your manuscript has been judged scientifically suitable for publication and will be formally accepted for publication once it meets all outstanding technical requirements.

Within one week, you’ll receive an e-mail detailing the required amendments. When these have been addressed, you’ll receive a formal acceptance letter and your manuscript will be scheduled for publication.

An invoice will be generated when your article is formally accepted. Please note, if your institution has a publishing partnership with PLOS and your article meets the relevant criteria, all or part of your publication costs will be covered. Please make sure your user information is up-to-date by logging into Editorial Manager at Editorial Manager® and clicking the ‘Update My Information' link at the top of the page. For questions related to billing, please contact billing support.

If your institution or institutions have a press office, please notify them about your upcoming paper to help maximize its impact. If they’ll be preparing press materials, please inform our press team as soon as possible -- no later than 48 hours after receiving the formal acceptance. Your manuscript will remain under strict press embargo until 2 pm Eastern Time on the date of publication. For more information, please contact onepress@plos.org.

Kind regards,

José Luiz Fernandes Vieira

Academic Editor

PLOS One

# Editor report: Acceptance letter

**DOI:** 10.1371/journal.pone.0351511.r004

**Authors:** José Luiz Vieira

PONE-D-26-11390R1

PLOS One

Dear Dr. Jeevaratnam,

I'm pleased to inform you that your manuscript has been deemed suitable for publication in PLOS One. Congratulations! Your manuscript is now being handed over to our production team.

At this stage, our production department will prepare your paper for publication. This includes ensuring the following:

* All references, tables, and figures are properly cited

* All relevant supporting information is included in the manuscript submission,

* There are no issues that prevent the paper from being properly typeset

You will receive further instructions from the production team, including instructions on how to review your proof when it is ready. Please keep in mind that we are working through a large volume of accepted articles, so please give us a few days to review your paper and let you know the next and final steps.

Lastly, if your institution or institutions have a press office, please let them know about your upcoming paper now to help maximize its impact. If they'll be preparing press materials, please inform our press team within the next 48 hours. Your manuscript will remain under strict press embargo until 2 pm Eastern Time on the date of publication. For more information, please contact onepress@plos.org.

You will receive an invoice from PLOS for your publication fee after your manuscript has reached the completed accept phase. If you receive an email requesting payment before acceptance or for any other service, this may be a phishing scheme. Learn how to identify phishing emails and protect your accounts at https://explore.plos.org/phishing.

If we can help with anything else, please email us at customercare@plos.org.

Thank you for submitting your work to PLOS One and supporting open access.

Kind regards,

PLOS ONE Editorial Office Staff

on behalf of

Dr. José Luiz Fernandes Vieira

Academic Editor

PLOS One
